# Lipid metabolism of hepatocyte-like cells supports intestinal tumor growth by promoting tracheogenesis

**DOI:** 10.1101/2025.04.04.647255

**Published:** 2025-04-05

**Authors:** K. Huang, T. Miao, E. Dantas, M. Han, Y. Hu, K. Wang, J. Sanford, M. Goncalves, N Perrimon

**Affiliations:** 1 Department of Genetics, Blavatnik Institute, Harvard Medical School, Harvard University, Boston, MA 02115, USA; 2 Howard Hughes Medical Institute, Boston, MA, USA; 3 Division of Endocrinology, Department of Medicine, Weill Cornell Medicine, New York, NY 10065, USA.; 4 Meyer Cancer Center, Weill Cornell Medicine, New York, NY 10065, USA.

## Abstract

Tumors require metabolic adaptations to support their rapid growth, but how they influence lipid metabolism in distant tissues remains poorly understood. Here, we uncover a novel mechanism by which gut tumors in adult flies reprogram lipid metabolism in distal hepatocyte-like cells, known as oenocytes, to promote tracheal development and tumor growth. We show that tumors secrete a PDGF/VEGF-like factor, Pvf1, that activates the TORC1-Hnf4 signaling pathway in oenocytes. This activation enhances the production of specific lipids, including very long-chain fatty acids and wax esters, that are required for tracheal growth surrounding the gut tumor. Importantly, reducing expression in oenocytes of either the transcription factor *Hnf4*, or the elongase mElo that generates very long chain fatty acid suppresses tumor growth, tracheogenesis, and associated organ wasting/cachexia-like phenotypes, while extending lifespan. We further demonstrate that this regulatory pathway is conserved in mammals, as VEGF-A stimulates lipid metabolism gene expression in human hepatocytes, and lung tumor-bearing mice show increased hepatic expression of *Hnf4* and the lipid elongation gene *Elovl7*. Our findings reveal a previously unrecognized tumor-host interaction where tumors non-autonomously reprogram distal lipid metabolism to support their growth. This study not only identifies a novel non-autonomous role of the TORC1-Hnf4 axis in lipid-mediated tumor progression but also highlights potential targets for therapeutic intervention in cancer-associated metabolic disorders.

## Introduction

Tumor growth demands a growing supply of nutrients and oxygen. The growing tumor constantly shapes its microenvironment to favor fast growth and expansion, through secreted factors and extracellular matrix. The composition of the tumor microenvironment (TME) varies between tumor types, but the hallmarks include increased blood vessels, immune cells, stromal cells, and extracellular matrix [1]. To overcome the increasingly hypoxic and acidic environment, TME promotes angiogenesis to restore oxygen and nutrient supply. In addition to modulating the local TME, malignant cells can exert systemic effects on distant organs—including the liver, skeletal muscle, and brain—to reshape the host’s metabolic pathways, ultimately promoting further tumor progression [2–6]. Liver is a metabolic center for amino acids, carbohydrates, and lipid. A key liver-specific metabolic function is the complete urea cycle (UC), which eliminates excess ammonia by converting into urea, a less toxic waste. Previous studies have identified evidence of decreased UC activity in the livers of 4T1 breast cancer bearing mice, and the plasma of children with cancer [5], demonstrating an existing interaction between tumors and the liver. Delineating molecular pathways involving tumor-host interaction can provide novel therapeutic targets.

Lipids play a critical role in cancer progression, by serving as building blocks, energy sources and signaling molecules. It has been observed that tumor cells reprogram lipid metabolism to enhance growth, characterized by increased lipid synthesis, lipid uptake, fatty acid oxidation (FAO) and lipid storage [7]. Tumor cells also actively reshape lipid metabolism in cells from the TME through secreted signaling molecules and drive TME to favor tumor growth. For example, increased lipid uptake and FAO in regulatory T cells (Tregs), tumor-associated macrophages (TAMs) and myeloid-derived suppressor cells (MDSCs), promotes their immunosuppressive function [8–10]. However, whether tumor cells also reshape lipid metabolism in distal organs, such as liver, which is a metabolic center for lipid transport, is not known.

To better understand the reciprocal communication between liver and tumor cells, we utilize *Drosophila* which has emerged as a simple yet powerful model to study tumor-host interactions [11]. The fly midgut, oenocytes, fat body and malpighian tubules are functionally equivalent to human digestive tract, hepatocytes, adipose tissue and kidney, respectively [12]. Oenocytes, which are hepatocyte-like cells, are important for mobilizing stored lipids during starvation and interact closely with metabolic tissues, such as the fat body to regulate lipid uptake and reprocessing [13, 14]. In addition to regulating lipid metabolism under nutritional deprivation, oenocytes are the major site for synthesis of cuticular hydrocarbons in pterygote insects [15–17]. Adult oenocytes synthesize hydrocarbons using fatty acyl-CoA precursors via reduction to fatty aldehyde and oxidative decarbonylation [18, 19]. Many fatty acid synthases, elongases and desaturases expressed in adult oenocytes produce various types of fatty acyl-CoA substrates that are processed to form a complex blend of cuticular hydrocarbons [20–23]. Hepatocyte nuclear factor 4 (Hnf4), an oenocyte enriched transcription factor, plays an important role in regulating the conversion of lipids to very long chain fatty acid, which are important substrates for hydrocarbon synthesis [23]. Oenocytes are the major site for lipid transport and processing, reminiscent of human hepatocytes. However, whether and how oenocytes regulate tumor growth is unknown.

The *Drosophila* trachea is a network of oxygen transporting tubes, the functional equivalent of mammalian blood vessels [24]. Trachea is composed of terminal tracheal cells (TTCs), which are highly plastic cells, and the terminal branches (TBs) or tracheoles which deliver oxygen to the tissues. TTCs are oxygen sensing cells, and hypoxic conditions promote the formation of TBs through hypoxia-inducible factor-1α (HIF-1α; Similar (Sima) in *Drosophila*), similar to the role of HIF-1α in mammalian angiogenesis. Trachea resides in proximity of many tissues, including the midgut. Trachea penetrate the visceral muscles to access the intestinal epithelium to supply it with oxygen [25]. The intestinal epithelium is apicobasally polarized, composed of intestinal stem cells (ISCs) and their progeny. Damage induced by enteric infection, oxidative agents and tumor growth can increase tracheogenesis on the midgut, which is necessary for efficient damage-induced ISC-mediated regeneration [26, 27]. Additionally, tumor-induced tracheogenesis in *Drosophila* is reminiscent of cancer-induced angiogenesis in mammals [26].

Using a Drosophila gut tumor model, we uncover a novel mechanism through which gut tumors reprogram lipid metabolism in distal oenocytes to promote both tracheal development and tumor growth. We find that tumors secrete a PDGF/VEGF-like factor, Pvf1, which activates the TORC1-Hnf4 signaling pathway in oenocytes. This activation boosts the production of specific lipids, including very long-chain fatty acids and wax esters, essential for tracheal growth around the tumor. Notably, reducing the activity of Hnf4 and mElo, an elongase involved in generating very long-chain fatty acids, in oenocytes suppress tumor growth, tracheal development, and cachexia-like phenotypes, while also extending lifespan. Additionally, we demonstrate that this regulatory pathway is conserved in mammals, as VEGF-A stimulates lipid metabolism gene expression in human hepatocytes, and tumor-bearing mice exhibit increased hepatic expression of lipid elongation genes. Our findings highlight a previously unrecognized tumor-host interaction, where tumors non-autonomously reprogram distal lipid metabolism to facilitate their growth.

## Results

### Oenocyte lipid metabolism regulated by Hnf4 controls tumor proliferation

To investigate how tumor growth rewires lipid metabolism of the whole fly, we expressed an active form of *Yorkie (yki*^*3SA*^), in adult gut stem cells, using the intestinal stem cells (ISCs)/enteroblast driver (*Esg-Gal4* or *Esg-LexA*), hereafter referred to as Yki flies [28]. *Esg-Gal4* or *Esg-LexA* was combined with the *tubulin* promoter-driven temperature-sensitive *GAL80 (tubP-GAL80*^*TS*^), to control the temporal expression of Gal4 or LexA. Next, we conducted untargeted lipidomic profiling using an LC-MS/MS-based platform of whole-body samples of adult female flies (*Esg-LexA* > *LexAop-yki*^*3SA*^) expressing *yki*^*3SA*^ for 3 days, together with control flies (*Esg* > +). The complete *Drosophila* genotype information can be found in Supplementary Table 1. Female flies are used in the analysis unless otherwise noted, because they show more significant and consistent tumor-associated cachexia phenotypes. We detected 2471 lipid species in total representing 45 lipid classes (Supplementary Table 2). The lipidomic analysis showed an increase in wax ester (WE), monogalactosyldiacylglycerol (MGDG), phosphatidylglycerol (PG), phosphatidylcholine (PC), and ceramide (Cer) level in Yki flies (Figure 1A). We also observed a slight reduction of triglycerides (TG) levels in Yki flies at early stage, in agreement with previous findings [29]. WEs are major components of cuticular lipids, which cover nearly all parts of terrestrial arthropods [30] to restrict water loss, and WE levels are significantly increased in Yki flies (Figure 1B), yet the significance of this regulation is unknown. As the biosynthesis of cuticular lipids mainly occurs in *Drosophila* oenocytes and Hnf4 is a main regulator of this process [23], we tested whether oenocyte Hnf4 regulates the level of WEs in wild type flies. WEs were reduced when *Hnf4* was knocked down in wild type adult oenocytes using the *PromE(800)-Gal4* Gene-switch driver (hereafter referred to as PromE^gs^) [14], where the driver is activated upon mifepristone feeding (RU) (Figure 1C). Next, to test whether Hnf4 regulates WE synthesis in Yki flies, we used the LexA-LexAop system to induce Yki tumors in the gut and the Gal4/UAS system combining GAL80 in oenocytes (*PromE-GAL4, Tub-GAL80*^*TS*^, referred to as *PromE*) to knockdown *Hnf4*. Knockdown of *Hnf4* in oenocytes decreased Yki-induced WE levels (Figure 1D), indicating that *Hnf4* expression in oenocytes is required for the increase of WE level in Yki flies.

As a nuclear receptor, the localization of Hnf4 is highly enriched in oenocyte nuclei compared to the fat body [23, 31]. To visualize the localization of Hnf4, we tagged the endogenous *Hnf4* gene with 127D01, a small epitope tag that can be detected with a specific nanobody [32, 33], using an homology directed repair pathway CRISPR-Cas9 targeted insertion method [32]. To validate the Hnf4-127D01 line, we knocked down *Hnf4* in oenocytes (Figure S1A), which significantly reduced its nuclear localization (Figure S1B). Hnf4-127D01 in wild type oenocytes showed high level of nuclear localization, consistent with previous findings (Figure 1E) [31]. Interestingly, increased nuclear localization of Hnf4-127D01 was observed in the oenocytes of Yki flies (Figure 1E–1F). Altogether, these data suggest that oenocytes in Yki flies have increased Hnf4 activity. To test whether oenocyte Hnf4 signaling is required for tumor growth, we measured ISC-mitosis using phospho-histone 3 (pH3+) staining. Inhibition of *Hnf4* in oenocytes of Yki flies *(Esg-LexA*>*yki*^*3SA*^*; PromE*>*Hnf4-i)* for 3 days showed a decrease in pH3+ staining compared to controls (*Esg*> *UAS-yki*^*3SA*^), and a slower growth rate for tumor (Figure 1G). Importantly, knockdown of *Hnf4* alone in wild type animals does not affect pH3+ number compared to control (Figure 1G), indicating that the role of Hnf4 in Yki flies relates to the aberrant regulation of Hnf4 in Yki flies. In wild type flies, knocking down *Hnf4* in oenocytes for 7 days resulted in a slight reduction in ovary size (Figure S1C–D), suggesting long-term reduction of *Hnf4* is detrimental to the ovary development. Further, oenocyte *Hnf4* knockdown in Yki flies for 3 days could rescue the level of both ovary wasting, bloating and induced wet weight (Figure 1H–1I, Figure S1E), indicating that aberrant Hnf4 activity in oenocytes contribute to tumor growth and associated cachexia phenotypes.

Our result suggests that Hnf4 transcriptional activity is induced in Yki oenocytes. To validate this, we measured whether the expression of Hnf4 targets in Yki oenocytes also increased. Published studies identify that Hnf4 target genes are involved in beta-oxidation and very long chain fatty acid (VLCFA) biosynthesis in oenocytes [23, 34], we tested whether VLCFA biosynthesis genes (*Hnf4, mElo, CG30008, CG16904, FarO, ACC, HDAC, TER, Cyp4g1, Cpr*), as well as genes involved in beta-oxidation (*Whd, Scu, Yip2* and *CG7461)* were dysregulated in Yki flies. *CG30008, CG16904, FarO, ACC, HDAC, TER, Cyp4g1, Cpr, Whd, Scu, Yip2* showed no difference between control and Yki flies (Figure S1F). However, we observed a significant increase in *mElo* (Figure 1K), which encodes a methyl-branched cuticular hydrocarbon elongase [21] and a minor increase for *Hnf4 and CG7461* (Figure S1F).

*mElo* has recently been shown to produce long chain methyl-branched cuticular hydrocarbons in oenocytes that help prevent desiccation [21], a key function of *Hnf4* in oenocytes [23]. Additionally, *mElo* expression is highly enriched in wild type adult oenocytes according to snRNAseq data (Figure 1L). Inhibition of *Hnf4* reduced *mElo* expression, and overexpression of *Hnf4* led to increased levels of *mElo* (Figure S1G–H), consistent with a previous report that mElo is a transcriptional target of *Hnf4* [23]. Further*, mElo* is highly induced in the oenocytes of Yki flies and this induction is blocked when *Hnf4* is knocked down (Figure 1K).

In wild type flies, reduction of *mElo* in oenocytes did not affect pH3+ number in the gut (Figure 1M). To test the function of oenocyte *mElo* during tumor growth, we reduced the level of oenocyte *mElo* in Yki flies and observed fewer pH3+ in the gut, suggesting that *mElo* contributes to Yki tumor growth (Figure 1M). We validated the effectiveness of *mElo* knockdown in the oenocytes (Figure S1I). Unlike *Hnf4,* reducing *mElo* expression in wild type oenocytes did not cause steatosis and a reduction of ovary size, suggesting that other targets of *Hnf4* regulate oenocytes steatosis and ovary size (Figure 1N and Figure S1J–S1K). Consistently, *mElo* knockdown did not rescue ovary wasting in Yki flies (Figure S1L).

To assess whether oenocyte lipid metabolism affects the viability of Yki flies, we inhibited *Hnf4* in oenocytes and observed a significant lifespan extension (Figure 1O). Inhibition of mElo in oenocytes of Yki flies also significantly increased lifespan (Figure 1P). Finally, *mElo* has a sex specific function in regulating different types of hydrocarbons [21]. Therefore, we tested whether *mElo* also regulates Yki viability in male flies. Strikingly, inhibition of *mElo* also significantly increased the lifespan in Yki males (Figure S1M).

### Oenocyte Hnf4 activity is regulated by TORC1

We next sought to determine the factors regulating Hnf4 level in oenocytes. Previous studies have shown that inhibiting the TORC1 pathway in oenocytes leads to steatosis [35], a phenotype that mimics reduced Hnf4 expression in these cells [23], suggesting that the TORC1 pathway positively regulates Hnf4 transcription in oenocytes. The Tuberous Sclerosis Complex (TSC) is known to negatively regulate TORC1 by inhibiting RAS homolog enriched in brain (Rheb) activity [36]. Using a previously published snRNAseq dataset [35], we found that overexpression of Tsc1 together with Tsc2 (TSC1,2) in oenocytes, which inhibits TORC1, resulted in reduced Hnf4 expression specifically in oenocytes (Figure 2A), but not in muscle nor fat body tissues, suggesting that that TORC1 positively regulates Hnf4 level in oenocytes.

Next, we examined the protein level of endogenously tagged Hnf4-127D01 following *TSC1,2* over-expression and observed a significant reduction in Hnf4-127D01 levels in oenocytes, particularly in the nucleus as Hnf4 is a transcription factor (Figure 2B–C). Given that Hnf4 regulates hydrocarbon synthesis in oenocytes and its knockdown increases the susceptibility to dry starvation [23], we overexpressed *TSC1,2* in oenocytes and found that it led to an increased sensitivity of flies to dry starvation (Figure S2A). Additionally, as Hnf4 regulates the expression of genes involved in very-long-chain fatty acid (VLCFA) synthesis [23], we analyzed the expression of VLCFA synthesis genes in snRNAseq data from control (*PromE*>*EGFP*) versus TSC1,2-overexprerssing oenocytes (*PromE*>*TSC1,2*). Using uniform Manifold Approximation and Projection (UMAP), which identifies cell types in mixed populations based on snRNAseq data, we found that VLCFA synthesis genes were highly enriched in oenocytes (Figure 2D). The ModuleScore, calculated by averaging the expression of the VLCFA synthesis pathway genes and subtracting the expression of a randomly selected gene set expression, decreased in oenocytes upon *TSC1,2* overexpression (Figure 2D).

Inhibition of TORC1 through overexpressing *TSC1,2,* or overexpressing Proline-rich Akt substrate 40 kDa (*PRAS40)*, or by feeding flies with rapamycin, all induced steatosis in oenocytes (Figure 2E and S2B–C), as observed following *Hnf4* knockdown in oenocytes (Figure 1N). However, simultaneous overexpression of *Hnf4* with *TSC1*,2 prevented the development of steatosis (Figure 2E), indicating that TORC1 regulates oenocyte lipid metabolism via Hnf4. Lastly, *TSC1,2* overexpression also reduced the expression of *mElo* and *FASN2,* which are Hnf4 target genes, and overexpressing *Hnf4* simultaneously blocked this decrease (Figure 2F–G). Together, these findings suggest that Hnf4 acts downstream of TORC1 to regulate lipid metabolism in oenocytes.

Next, we also tested the effects of oenocyte *TSC1,2* in tumor growth. Consistent with *Hnf4* and *mElo* knockdown, *TSC1,2* overexpression in oenocytes reduced tumor induced WE levels (Figure 2H). We also observed an increased level of TORC1 activity, as measured by phospho-4EBP (p4EBP) staining in oenocytes of Yki flies (Figure 2I–J). Additionally, overexpressing *TSC1,2* blocked *mElo* expression in oenocytes of Yki flies (Figure 2K), Yki-induced pH3^+^ cells in the gut (Figure 2L), as well as Yki-induced ovary wasting and bloating (Figure 2M and S2D). Altogether, these results indicate that TORC1 regulate Hnf4 transcription and protein level in oenocytes and its activation is required for tumor growth.

### ISCs secreted Pvf1 regulates Hnf4 through PvR signaling in oenocytes and affects tumor associated phenotypes

TORC1 activity is regulated through various pathways, one of which includes PDGF/VEGF signaling. Studies have shown that PvR activation can stimulate the TOR pathway [35, 37, 38]. Consistent with this, muscle-secreted Pvf1 can regulate TORC1 activity in the oenocytes [35], suggesting that the activation of the TORC1-Hnf4 axis in oenocytes of Yki flies may occur through PvR activation. Yki-expressing ISCs are capable of secreting higher levels of the Pvf1 ligand, which activates PvR signaling in surrounding tissues [39]. However, *Pvf1* is expressed at low levels in oenocytes and is not significantly induced in Yki flies (Figure S3A–C). To test whether ISC secreted Pvf1 can induce oenocyte Hnf4 levels, we overexpressed *Pvf1* specifically in adult ISCs (*Esg*>*Pvf1*) and measured Hnf4-127D01 nuclear level in oenocytes. *Pvf1* overexpression in ISCs increased nuclear Hnf4-127D01 levels (Figure 3A–B), suggesting an increased transcriptional activity as measured by increased level of *mElo* expression (Figure 3C). To test whether ISC-derived Pvf1 activates PvR in oenocytes, we overexpressed an active form of PvR in oenocytes which was associated with increased nuclear Hnf4-127D01 level, as well as increased level of *mElo* expression (Figure 3D–F). To determine whether tumor secreted Pvf1 non-autonomously activates Hnf4 level in oenocytes, we knocked down *Pvf1* expression in the ISCs of Yki flies (*Esg*>*yki*^*3SA*^*; Pvf1-i*) and observed its effect on Hnf4-127D01 nuclear level. *Esg*>*yki*^*3SA*^*; Pvf1-i* significantly reduced the level of Hnf4-127D01 nuclear localization compared to *Yki* flies (*Esg*>*yki*^*3SA*^*; Luc-i)* (Figure 3G–H). Consistently, *mElo* expression was reduced in *Esg*>*yki*^*3SA*^*; Pvf1-i* flies compared to Yki flies (Figure 3I). We also tested whether Pvf1 reduction in Yki flies would affect tumor development, like Hnf4 inhibition in oenocytes. As reported previously [39], inhibition of *Pvf1* in the tumor gut rescues both bloating and ovary wasting (Figure 3J–K). Gut tumors in *Esg*>*yki*^*3SA*^*;Pvf1-i* flies exhibited slower growth at early stage, as shown by reduced number of pH3+ cells (Figure 3L).

Finally, to demonstrate that Pvf1 secreted from Yki tumors directly controls *mElo* expression through PvR, we used the dual Gal4 and LexA systems to reduce *PvR* level in oenocytes while inducing Yki in ISCs. *Esg-LexA*>*yki*^*3SA*^*;PromE*>*PvR-i* animals showed reduced *mElo* expression (Figure 3M), decreased the level of bloating and ovary wasting, reduced the number of pH3+ cells in gut tumors (Figure 3N–P), and extended lifespan, compared to Yki flies (Figure 3Q). Altogether, these data suggests that PDGF/VEGF induced by Yki tumor non-autonomously increases Hnf4 activity in oenocytes through PvR.

### Circulating lipids target trachea but not ISCs to promote trachea growth

To identify the lipid species induced by oenocytes in Yki flies, we performed lipidomic analysis on the fly hemolymph of control flies, *Esg-LexA*> *yki*^*3SA*^ and *Esg-LexA*> *yki*^*3SA*^*; PromE*>*Hnf4-i* flies (Figure 4A, Supplementary Table 3). Among the lipid species that are induced by Yki and decreased by *Esg-LexA* > *yki*^*3SA*^*; PromE*>*Hnf4-i*, the majority are phospholipids including phosphotidyl choline (PC), phosphatidylglycerol (PG) and phosphatidylethanolamine (PE), triglycerides (TGs) and acylcarnitines (AcCas). More specifically, AcCa 24:1 and AcCa 24:2, which are long-chain acylcarnitines (C≥12) secreted by mitochondria when metabolism is inhibited [40], were elevated in Yki flies while reduced by *Hnf4* knockdown (Figure 4B–C). Circulating PC 36:4, and PE 32:5 phospholipids and TG(18:3_16:0_16:1), TG(18:2_16:0_18:0) triglycerides were induced in Yki flies and rescued by *Hnf4* knockdown (Figure 4D–G). Interestingly, PC 36:4 is elevated in the serum and plasma samples from breast cancer patients [41] and TG(18:2_16:0_18:0) is induced in bladder cancer patients [42].

PC and PE are the most abundant phospholipids of cellular membranes and play important role in lipoprotein metabolism. In insects, lipid transport in circulation relies on lipophorins, which are the major lipoproteins. The increased circulation of PC 36:4 and PE 32:5 in Yki flies suggests an increase in lipoproteins that transport lipids from oenocytes towards trachea or the midgut.

The transport of lipids, such as phospholipids from oenocytes, to the trachea or ISCs to promote tumor growth has not been reported [26]. Thus, we inhibited Hnf4 activity in oenocytes by overexpressing *TSC1,2*, knocking down or overexpressing *Hnf4*, and observed no significant effect on the number of pH3+ cells in the midgut (Figure 4H). Since 95% of the hemolymph lipids are transported through lipophorin (Lpp) in flies [43], and that low-density lipoprotein (LDL) receptor homologues LpR1 and LpR2 promote Lpp internalization [44], we reduced LpR1 or LpR2 level in the ISCs. However, this had no impact on the trachea area, skeleton length, and only a minor increase in the number of branches in the *LpR1* knockdown condition (Figure S4A–C). Reducing LpR1 and LpR2 activities in the ISCs also did not affect the proliferation (Figure S4D). To further examine the role of LpR1 or LpR2 in ISC tumors, we reduced LpR1 and LpR2 levels while overexpressing Yki (*Esg*> *yki*^*3SA*^*;LpR1-i and Esg*> *yki*^*3SA*^*;LpR2-i*), and found that these manipulations did not impair Yki-induced tracheal growth (Figure 4I–K) or prevent Yki-induced ISC proliferation (Figure 4L). These results suggest that ISC proliferation does not rely on circulating lipids in the hemolymph.

We further tested whether disrupting *LpR1* and *LpR2* activities in trachea could blunt tracheal growth. Knocking down *LpR1* using a temperature sensitive trachea-specific driver (*btl-GAL4, UAS-srcGFP, Tub-GAL80*^*TS*^, referred to as *Btl*) reduced tracheal number of branches, tube area and skeleton length compared to control flies (*Btl*>+), whereas knocking down *LpR2* in trachea did not have a significant effect (Figure 4M–O). Reducing LpR1 and LpR2 activities in the trachea did not affect ISC proliferation (Figure S4E). This suggests that trachea growth, but not ISC growth, requires lipids generated from oenocytes transported through lipophorins.

### Oenocyte Hnf4 and mElo regulates tracheogenesis in the midgut

Next, we aimed to understand whether oenocyte lipid metabolism controls trachea growth in wild type guts. Consistent with Hnf4 knockdown, oenocyte specific *mElo* reduction and *TSC1,2* overexpression showed reduced level of whole body WE in non-Yki flies (Figure 5A–B). WEs are essential parts of the outer layer of the trachea [45–48], and *waterproof* (*wat*), which is involved in biosynthesis of wax, has been implicated in hydrophobic tracheal coating in *Drosophila* embryos [48]. Knocking down *Hnf4* did not affect the transepithelial barrier mediated by septate junctions in the midgut trachea on the midgut, as revealed by red dextran injection experiment (Figure 5C). However, reduction of oenocyte *Hnf4* altered the morphology of trachea associated with the midgut (R2-R4 regions) (Figure 5D). Specifically, we observed a reduction in the number of branches and total skeleton length, normalized to the area of the gut surface (Figure 5E–G). Consistently, we also observed a reduction in branch numbers, tube area and skeleton length when *mElo* was knocked down in oenocytes of wild type flies (Figure 5H–J). The effect of oenocyte Hnf4 on trachea number is not specific to the midgut, as we also observed a reduction of trachea total tube area in the ovary of wild type flies (Figure S5A–D). Overexpressing *Hnf4* or *mElo* is sufficient to induce total tracheal tube area and skeleton length in the midguts of wild type flies (Figure 5K–P). Finally, to eliminate the effect of RU feeding on trachea morphology, we fed control (*PromE*>*ctrl*) flies and observed no difference in trachea morphology (Figure S5E–G).

### Hnf4-mElo axis in oenocytes mediates lipid metabolism and tracheogenesis induced by Yki tumors

Tumor growth in the adult *Drosophila* midgut has been previously shown to promote tracheole density [26]. Similarly, we observed an increase in the number of trachea branches, total tube area, and total tube length in the surrounding tissue of the Yki midgut (Figure S6A–D). This led us to investigate whether oenocyte lipid metabolism mediated by *Hnf4-mElo* axis is crucial for trachea growth in the Yki midgut. Our lipidomic analysis showed reduced whole body WE level when *Hnf4* is knocked down, or when *TSC1*,2 is overexpressed in the oenocytes of Yki flies (Figure S6E).

To explore whether perturbations of lipid metabolism in oenocytes could block Yki-induced tracheogenesis in the midgut, we manipulated lipid metabolism by overexpressing TSC1,2 or knocking down Hnf4 or mElo. Using the LexA/LexAop and Gal4/UAS systems, we found that overexpressing TSC1,2 or reducing mElo significantly decreased the number of tracheal branches, total tube area, and total tube length in the Yki midgut. Reducing Hnf4 levels, on the other hand, only decreased the total tube area (Figure 6A–C).

In addition, to verify whether midgut secreted Pvf1 regulates tracheal growth, we knocked down *Pvf1* in ISC in addition to Yki overexpression. Reduction of *Pvf1* blocked Yki induced number of branches, tube area and skeleton length (Figure 6D–F), indicating that oenocyte lipid metabolism is important for trachea growth induced by Yki.

### Regulation of hepatocyte lipid metabolism by pro-angiogenic factors in tumor models

**Figure 7:**
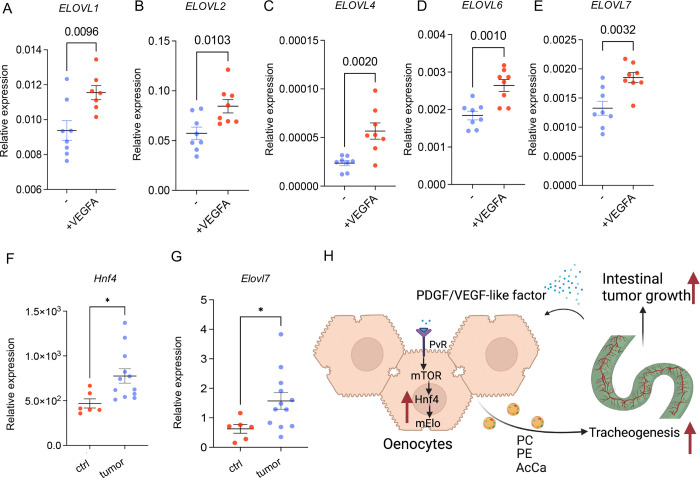
Regulation of hepatocyte lipid metabolism by pro-angiogenic factors in tumor models. (A-E) Relative expression levels of *ELOVL* fatty acid elongase 1–7 in HepG2 cells incubated with vascular endothelial growth factor A (VEGF-A) for 24 hours, compared to controls. N = 4 biological replicates, from 2 independent experiments. (F-G) Relative expression levels of *Hnf4a* and *Elovl7* in liver samples from *KrasG12D/+;Lkb1f/f* (KL) tumor-bearing mice compared to control mice. N = 4 biological replicates, from 2 independent experiments. (H) Schematic representation of the proposed mechanism, depicting tumor-secreted PDGF/VEGF-like factors activating the mTOR-Hnf4 axis in hepatocyte-like oenocytes to promote lipid synthesis, which are then transported to trachea. Dashed lines indicate hepatocyte regions or cells analyzed. Data are presented as mean ± s.e.m.; statistical analysis was performed using Student’s t-test.

Using the *Drosophila* Yki tumor model, we found that the tumor secreted PDGF/VEGF-like factor Pvf1 can non-autonomously regulate Hnf4 activity in hepatocyte-like cells to promote the production of VLCFA or related lipids. To test whether this mechanism is conserved in mammals, we incubated HepG2 cells with vascular endothelial growth factor A (VEGF-A), which is orthologous to Pvf1 in flies and a potent pro-angiogenic growth factor expressed in many types of tumors. We measure the expression level of ELOVL fatty acid elongase 1 – 7 (*ELOVL1–7*). Incubating HepG2 with VEGF-A for 24 hours can significantly increase the level of *ELOVL1, 2, 4, 6* and *7*, suggesting a conserved regulation of PDGF/VEGF on lipid metabolism in hepatocytes (Figure 7A–E). In addition, we collected liver samples from *KrasG12D/+;Lkb1f/f* (KL) mice, in which tumors were specifically induced in the lungs, and analyzed the expression levels of *Hnf4a* and *Elovl1–7* in the hepatocytes. *Hnf4a* and *Elovl7* showed higher expression level in the tumor bearing mice, compared to control, suggesting that tumor growth in the lung increased the level of genes involved in lipid metabolism in distal hepatocyte tissues in the KL mouse model (Figure 7F–G). Altogether, these data showed that a conserved mechanism of PDGF/VEGF-like factor in regulating VLCFA metabolism in hepatocytes through Hnf4.

## Discussion

We reveal a novel mechanism by which gut tumors in Drosophila reprogram lipid metabolism in oenocytes to promote tracheal development and tumor growth. Our findings demonstrate that tumors secrete a PDGF/VEGF-like factor, Pvf1, which activates TORC1 activity, triggering the expression of mElo through Hnf4 in oenocytes. This TORC1-Hnf4 signaling pathway enhances the production of specific lipids, such as very long-chain fatty acids and wax esters, which stimulate tracheal growth, a crucial process for tumor expansion. We also show that reducing Hnf4 or mElo activity in oenocytes suppresses tumor growth, tracheogenesis, and tumor-induced cachexia, while extending lifespan. Additionally, we highlight the conservation of this regulatory pathway in mammals, where VEGF-A stimulates lipid metabolism gene expression in human hepatocytes. Furthermore, lung tumor-bearing mice exhibit increased Hnf4 and Elovl7 expression in peripheral hepatocytes.

Pvf1, a tumor-secreted factor, has previously been linked to tumor-induced muscle and fat body wasting, as well as renal dysfunction in *Drosophila* [39, 49, 50]. In this study, we uncover a novel role for tumor-secreted Pvf1 in modulating oenocyte lipid metabolism through TORC1 and Hnf4, which regulates the expression of the oenocyte-enriched gene mElo (Figure 3). While Hnf4 activity is known to be induced by starvation and during the developmental transition from pupae to larvae [23, 34], its upstream regulators remain poorly understood. In MEF cells, the Hnf4 binding motif is highly enriched in the promoters of TORC1-induced genes [51], suggesting Hnf4α mediates the transcriptional activities of TORC1. Here, we demonstrate that TORC1 positively regulates Hnf4 protein levels in the oenocyte nucleus through transcriptional regulation (Figure 2A). However, the precise mechanisms controlling *Hnf4* transcription downstream of TORC1 remain unclear. REPTOR and REPTOR-BP are likely key players in this process, as they are the major effectors of the transcriptional response induced by TORC1 inhibition [52].

Our study identified a novel mode of communication between oenocytes and the gut. Oenocytes produce lipid substrates that support gut tracheal growth, a process crucial for tumor expansion and potentially ISC growth. Similar to tumor, bacterial infections and epithelial damage also trigger ISC proliferation [53–55], suggesting that oenocyte-secreted lipids may also support stress-induced ISC expansion. We found that lipids from oenocytes are acquired by the tracheal cells instead of ISCs, as knockdowns of *LpR1* and *LpR2* in ISCs have no effects in tumor growth (Figure 5I–L). In contrast, reducing *LpR1* in tracheal cells was sufficient to decrease tracheal development (Figure 5M–O). This may be due to the trachea’s direct contact with hemolymph, allowing it to actively acquire lipids, while the midgut is covered by visceral muscle and basement membrane [56, 57].

The mechanism by which VLCFA metabolism regulates tracheogenesis remains unclear, though it may involve the induction of Reactive Oxygen Species (ROS) in the trachea. ROS has been implicated in the activation of HIF-1α/Sima, and that VLCFAs is known to induce mitochondrial dysfunction and ROS production [58–60]. Increased ROS levels, through treatments such as paraquat, H_2_O_2_ or bacterial infection, have been shown to induce tracheogenesis in the midgut [26]. Furthermore, manipulating ROS level in the trachea alone can trigger tracheal branching and ISC proliferation [26]. Interestingly, overexpressing *Hnf4* alone in oenocytes could promotes tracheogenesis, but does not significantly induce ISC proliferation (Figure 4H). This difference may be attributed to variations in driver strength, with tracheal development occurring prior to ISC proliferation.

We observed that VEGF-A stimulates lipid metabolism gene expression in human hepatocytes, and lung tumor-bearing mice showed increased expression of *Hnf4* and *Elovl7* in peripheral hepatocytes. This work provides new insights into tumor-host interactions, challenging the conventional view that tumor-associated metabolic changes are merely passive processes and instead demonstrating their role in promoting tumor growth. Specifically, inhibiting *Hnf4* and *mElo* in oenocytes slows tumor growth, halts tumor-induced cachexia, and extends the lifespan of flies (Figures 1N, 1P, and S1M). Yki activation increased levels of AcCas, PCs, and TAGs in the hemolymph, and inhibiting *Hnf4* in oenocytes reversed these lipid changes induced by Yki (Figure 4A). This study identifies the TORC1-Hnf4-Elovl7 axis and tumor-induced lipid metabolism as potential therapeutic targets for managing tumor growth or as biomarkers for early tumor detection.

Hnf4 is a master regulator of multiple metabolic pathways in *Drosophila* and inhibiting it could have broad effects on the host. For instance, knocking down *Hnf4* in non-tumor-bearing flies led to increased steatosis and smaller ovaries (Figures 1N, S1C–D). These wide-ranging effects may limit the efficacy of Hnf4 inhibition as an anti-tumor strategy. In contrast, mElo, which is more specifically involved in lipid elongation, presents a more targeted inhibition with lower toxicity and a greater extension of lifespan (Figures 1P, S1M). Our study also suggests a potential role for elongases in tumor-induced angiogenesis, as overexpression of ELOVL7 has been linked to prostate cancer and is required for cancer growth [61]. While mElo is exclusively expressed in oenocytes in flies (Figure 1L), its orthologue, ELOVL7, is widely expressed in multiple tissues, including the prostate, kidney, brain, small intestine, and thyroid [62]. It is plausible that ELOVL7 is upregulated in various tissues in cancer patients, indicating additional tissue targets for anti-angiogenic therapies.

In summary, our data reveal a novel paradigm in which growing tumors actively acquire substrates for tracheogenesis by secreting a PDGF/VEGF-like factor that activates the TORC1-Hnf4 axis in distal oenocytes, inducing the expression of mElo. This promotes the synthesis of VLCFAs, which are transported to the trachea to further support tumor growth (Figure 7H).

## Materials and Methods

### Drosophila stocks and husbandry

All experiments were conducted using female flies due to their more consistent bloating phenotype and larger oenocyte, which facilitated dissection. For temperature-sensitive experiments, fly crosses were set up and maintained under a 12:12 hour light:dark cycle at 18°C. Crosses were discarded after 5 days to maintain consistent population density. Following eclosion, adult flies were collected and kept at 18°C for 2–3 days. GAL80^TS^ driver activation was achieved by incubating the flies at 29°C for the durations specified in each experiment. For experiments involving the GeneSwitch driver, fly crosses were maintained at 25°C under a 12:12 hour light:dark cycle and discarded after 3 days. Adult progenies were collected and fed on food containing 200 μM RU486 (Mifepristone, Cayman Chemicals, 10006317), prepared by dissolving the compound in 95% ethanol before adding it to standard fly food. Flies were fed on RU486-containing food for 7 days.

Esg^ts^ (*esg-Gal4, UAS-GFP, tub-GAL80*^*TS*^), *Esg-LexA* and *LexAop-yki*^*3SA*^-*GFP, Attp40 UAS-Luciferase* are from the Perrimon lab stock collection.

The following strains were obtained from the Bloomington *Drosophila* Stock Center (BL), Vienna Drosophila Resource Center (V) and flyORF (F): *UAS-Hnf4 RNAi* (BL# 29375), *UAS-mElo RNAi* (BL# 44510), *UAS-yki*^*3SA*^ (BL#28817), *UAS-Pvf1-3xHA* (F002862), *UAS-PvR[A]* (BL#58496), *UAS-Pvf1 RNAi* (V#102699), *UAS-PvR RNAi* (V#43459), *UAS-Luciferase RNAi* (BL#31603), Oe^ts^ (*Desat1-Gal4, tubP-Gal80*) (BL# 65406), *UAS-LpR1 RNAi* (BL# 50737), *UAS-LpR2 RNAi* (BL# 54461).

*UAS-Hnf4* is a gift from Dr. Carl Thummel [31]; *UAS-TSC1, TSC2* is a gift from Dr. Christen K Mirth [63] and UAS-PRAS40 was a gift from Dr. Aurelio Teleman [64], Btl^TS^ (*Btl-Gal4, UAS-srcGFP*) and *QF6* >*QUAS-mtdTomato* was a kind gift from Dr. Chrysoula Pitsouli [26]. PromE^GS^(*PromE-GeneSwitch-Gal4*) was a gift from Dr. Heinrich Jasper [65]. *UAS-CG18609* and *mFas-Gal4* were gifts from Dr. Henry Chung [66, 67]. *UAS-PRAS40* is a gift from Dr. Aurelio A Teleman [52].

Hnf4-127D01 was generated through CRISPR-mediated tagging of endogenous Hnf4 with 127D01-tag as described previously [32]. Briefly, we cloned the sequence 1 kb upstream and 1 kb downstream of the stop codon into the donor vector pScarlessHD-C-3×127D01-DsRed (Addgene#171578). A sgRNA plasmid pCFD3-Hnf4-sgRNA that target the seed sequence (CCAGAGACTGGTTACTAGAAGAA) near the stop codon of Hnf4 was injected into yw;nos-cas9/CyO embryos together with the donor plasmid. Positive transformants with red eye fluorescent eyes were outcrossed and successful Kis were confirms by PCR (F: GTACAACCGGAGCGAGGGTA; R: CAAAATGGTTCAAGGCATAACA) and sequencing. Subsequently, 3xP3dsRed was excised using piggyBac transposase (BL#8285).

### Untargeted lipidomic analysis

Lipidomics and metabolomics of fly samples was performed following the Folch method (Folch et al., 1957). 15 adult flies, or hemolymph from 200–300 adult flies were used for lipidomics and metabolomics. Solid samples were homogenized in a 1 mL Dounce homogenizer (TIGHT) on ice with 0.6 mL chloroform and 0.3 mL methanol (2:1 chloroform-methanol mixture). Homogenized samples were transferred to a 15 mL glass tube with a Teflon cap, and an additional 0.6 mL chloroform and 0.3 mL methanol were added to make the final volume approximately 1.8 mL. Hemolymph was transferred to a 15 mL glass tube containing 1.2 mL chloroform and 0.6 mL methanol (2:1 chloroform-methanol mixture). Samples were vortexed briefly and incubated at room temperature on a rotator for 30–45 minutes. Following incubation, 0.2 volumes (0.36 mL) of HPLC-grade deionized water (HPLC dH2O) were added. The mixture was vortexed three times, followed by centrifugation at 1000 × g for 10 minutes at 4°.

The upper aqueous phase, containing polar metabolites, was carefully collected using a glass pipette, while avoiding the middle phase. Metabolites were transferred to microcentrifuge tubes, evaporated under vacuum using a SpeedVac rotary evaporator overnight, and stored at −80°C. Polar metabolites were resuspended in 20 μL of HPLC-grade water and then injected and analyzed using a hybrid 6500 QTRAP triple quadrupole mass spectrometer (AB/SCIEX) connected to a Prominence UFLC HPLC system (Shimadzu). The lower phase, containing non-polar lipids, was transferred to a clean 1.85 mL glass test tube using a glass pipette. The phase was dried under nitrogen gas for approximately 30 minutes, and dried lipids were stored at −80°C to prevent oxidation. Non-polar lipids were resuspended in 35 μL of HPLC-grade 50% methanol/50% isopropyl alcohol (IPA) and then injected and analyzed using untargeted high-resolution LC-MS/MS on a Thermo QExactive Plus Orbitrap mass spectrometer. LipidSearch version 4.2 (Thermo Scientific) was used to identify lipid molecules, with the quantification of ion intensity by measuring the area size of identified peaks.

The full names of the lipid species can be found in Supplementary Table 4. For lipidomic quantification using whole fly bodies, the same number of flies was used for lipid extraction. Lipid species were normalized to the total reads in each sample. For hemolymph lipidomic analysis, lipid species were normalized to both the volume of hemolymph extracted and the total reads in each sample.

### RNA extraction Quantitative RT-PCR

Adult female fly oenocytes were dissected in cold 1xPBS before RNA extraction. For oenocyte dissection, the fat body was removed through liposuction and then oenocytes were removed from the cuticle using a fine-tip glass needle. Tissue lysis, RNA extraction, and cDNA synthesis were performed using Cells-to-CT Kit (Thermo Scientific, #44-029-53). For female adult whole body mRNA extraction, four flies per biological replicate were collected. Total RNA was extracted using Direct-zol RNA MicroPrep kit (Zymo Research, #R2060). mRNA was extracted from HepG2 cells using Directo-zol RNA MicroPrep kit (Zymo Research, #R2060). 30–50mg of mice livers were dissected and total RNA was extracted using Qiagen RNeasy Mini kit (Qiagen #74104). All the cDNAs were synthesized using the iScript cDNA synthesis kit (Bio-Rad, #1708896) in a 40ul reaction mixture using 1000ng total RNA. Quantitative real-time RT-PCR (RT-qPCR) assays were performed using iQ SYBR Green Supermix (Bio-Rad, #1708880) on a CFX96 Real-Time PCR Detection system (Bio-Rad). Two to four independent biological replicates were performed with two technical replicates. The mRNA abundance of each gene was normalized to the expression of *RpL32* or *αTub84B* for fly samples, Gapdh for mouse samples and ACTB for HepG2 samples. Primer sequences are listed in the Supplementary Table 5.

### Fly imaging

*Drosophila* ovaries were dissected in cold PBS. ovaries were imaged immediately in cold PBS using a ZEISS Axiozoom V16 fluorescence microscope. Flies were anesthetized on CO_2_ pad and imaged using ZEISS Axiozoom V16 fluorescence microscope. The size of the ovary and abdomen is measured using Fiji (version: 2.14.0/1.54f).

### Oil Red O staining

The method was as previously described [13]. Flies were dissected in PBS and fat bodies were removed with liposuction. Specimens were fixed in 4% paraformaldehyde for 20 minutes. Specimens were rinsed twice with distilled water. Oil Red O (O0625-, Sigma-Aldrich) was dissolved in isopropanol to make 0.1% Oil Red O stock. A working solution was prepared by combining 3 mL of 0.1% Oil Red O in isopropanol with 2 mL of distilled water. The solution was freshly prepared and filtered through a 0.2-μm syringe filter before use. Specimens were incubated for 25 minutes in Oil Red O stain, rinsed briefly with PBS and mounted in Vector Shield for imaging. Oil Red O images were taken using Olympus VS200 Slide Scanner, equipped with color camera (2448×2048 pixels, 3.45μm).

### Immunostaining and image analysis

For immunostaining using the nanobodies, fly abdomens were dissected in PBS at room temperature with fat bodies removed and fixed in 4% formaldehyde in PBS for 30 minutes. Specimens were washed three times in PBST (0.1% Triton-X-100), 5 minutes each. The specimens were then incubated in 5% NGS (normal goat serum in PBST) for 1 hour. Nb127D01-ALFA, 0.2mg/ml was used at a dilution of 1:500, incubating the samples for 1 hour and a half with mild shaking. Followed by 0.1% PBST washes, 5 minutes each wash. AlexaFluor 594 anti-Alpaca (Jackson Immuno Research, 128-585-232) was diluted 1:400 in PBS-TX + 5% NGS and incubate with the specimens at room temperature with mild shaking for 1 hours. After washes and DAPI staining, tissues were mounted in Vectashield and imaged with Olympus VS200 Slide Scanner or confocal microscope Zeiss Axio Observer Z1.

For regular immunostainings, fly tissues were dissected in PBS at room temperature. Tissues were fixed in 4% paraformaldehyde in PBS for 30 minutes. Specimens were washed three times in 0.1% PBST and incubated in 5% NDS solution for 1 hour. Tissues were then incubated with primary antibodies diluted in PBST and blocking solution overnight at 4°. Then the tissues were washed three times with PBST at room temperature. Secondary antibodies were applied for 1 hour. Excess antibodies were washed in PBST, and tissues were incubated with DAPI (Invitrogen, D1306) dilute in PBST for 5 minutes. Tissues were then mounted in Vectashield (Vector Laboratories Inc, H-1000). The following primary antibodies were used: rabbit-anti-pH3 (1:500, Cell Signaling, 9701), rabbit-anti-p4E-BP (1:800, Cell Signaling, 2855S). Secondary antibodies against mouse, rabbit, chicken or alpaca conjugated to Alexa Fluor 488 and 594 (Jackson Immuno Research) were used at 1:800 for anti-p-4EBP, 1:1000 for anti-pH3 and 1:400 for Nb127D01-ALFA.

### Lifespan analysis

For survival analysis, flies were collected within 24 hours post-eclosion (20 females and 3 males per vial) and maintained on standard laboratory fly food at 18°C, with 60% humidity, and a 12-hour light/dark cycle. After two days, mature and mated females were transferred to 29°C to induce gene expression. To maintain vial cleanliness, flies were transferred to fresh vials every two days, and the number of dead flies was recorded daily.

### snRNAseq analysis

Data and plots were generated from the Fly Cell Atlas, generated using the 10X platform, “Stringent All” dataset. Other snRNAseq dataset was analyzed as described in [35], where a resolution of 0.4 was chosen as the clustering parameter.

For the Seurat Module Score analysis, Seurat AddModuleScore function was used to calculate the average expression of a group of genes in Very Long Chain Fatty Acid Synthesis pathway in each cell (for details of the list, see Supplementary Table 6), then subtracted by a randomly picked gene set expression.

### Rapamycin feeding

Rapamycin was dissolved in DMSO to make 2mM rapamycin stock. Rapamycin stock was further diluted to 200μM in ethanol and evenly distributed on to food surface. A small piece of Kimwipe was used to remove excess liquids on the food. DMSO dissolved in ethanol was used as control. Flies were transferred to new food every two days. Flies were fed for 6–7 days before dissection.

### Dextran injection

Using a fine glass needle, ~100nl of 50 mg/ml 10,000 MW TR-dextran (Thermo Fisher Scientific, D-1817) diluted in water was injected in the abdomen of adult females. 6 hours after injection, abdomen and thoraces were separated and whole abdomen were fixed in 4% paraformaldehyde for 30 minutes with mild shaking. After fixation, midguts were dissected and washed briefly with PBS and mounted in Vectashield. Midguts were imaged using a single point laser scanning confocal, Olympus IX83 to measure the trachea leakage. A Z-stack was taken, and maximum projections were used to generate final image for analysis.

### Trachea imaging

Generating fly strains that incorporate both a trachea reporter and other genetic modifications is challenging. Therefore, the following method, as described in [26], was used to quantify the trachea. Flies were dissected in cold PBS, and intestines were carefully extracted to maintain their original structure. Tissues were then fixed in 4% paraformaldehyde for 20 minutes, mounted in Vectashield, and imaged within 3 days. Whole intestine images were captured using an Olympus VS200 Slide Scanner equipped with a color camera. Only the upper layer of the tissue, in contact with the coverslip, was analyzed.

### Trachea analysis

To automate trachea measurement, we used the previously developed software AutoTube [68]. Only the topmost Z-stack, which provided the clearest image of the trachea, was analyzed. Images corresponding to the R2-R4 regions of the gut were cropped, avoiding areas with the major trunk of the trachea to prevent skewing the results. Images were thresholded on a white background and analyzed with AutoTube using the following settings:

- Input Color Channel: Green
- Intensity adjustment: Autocontrast
- Illumination correction: Illumination size set to 51
- Noise correction: BM3D
- Tube detection: Finer tubes with a tube size of 3
- Small regions removed: 0.01% of Ima
- Hole filling: Hole size set to 1
- Threshold type: MultiOtsu
- Short ramifications removed: Spur length set to 15
- Spatial branch merging: Spatial distance set to 10

The analysis provided results for “Number of Branches (μm),” “Total Tube Area (μm),” and “Total Skeleton Length (μm),” which were normalized to the corresponding intestinal area and were multiplied by an arbitrary number 1000000. Results from the R2-R4 regions were averaged for each gut.

### Animal Care and Tumor Induction

*Kras*^*G12D*^*/+;Lkb1*^*f/f*^ mice, previously described [3, 69], were backcrossed to the FVB strain. Male mice were maintained on a 12-hour light/dark cycle at an ambient temperature of 22°C, with ad libitum access to rodent chow (PicoLab Rodent 5053; Lab Diet, 3.43 kcal/g) and drinking water. Tumors were induced in adult male and female mice (12 to 20 weeks old) via intranasal administration of 75 μL PBS containing 2.5 × 10^7^ pfu of adenovirus SMV-CRE (Ad5CMV-Cre) purchased from the University of Iowa Gene Transfer Vector Core (Iowa City, IA). All animal experiments were conducted with the approval of the Institutional Animal Care and Use Committee (IACUC) at Weill Cornell Medicine (WCM) under protocol number 2013-0116.

### Mice tissue collection

Male mice were euthanized with CO_2_, followed by blood collection via cardiac puncture. Liver, gonadal adipose tissue, and skeletal muscles were dissected and flash-frozen in liquid nitrogen and stored at −80°. The control mice were a mix of fasted and fed mice. “Fasted” mice were fasted for 12 hours and “fed” mice were fasted for 12 hours and then allowed free access to food for 4 hours before tissue collection.

### VEGFA incubation

HepG2 cells were cultured in HyClone Dulbecco’s Modified Eagle Medium (DMEM) with high glucose (SH30022.01): with L-glutamine; without sodium pyruvate. Additional 50U penicillin/ml and 50ug streptomycin/ml were added. HepG2 cells were seeded at density of 5 × 10^5^ in 12-well-plate (VWR, 29442-040), treated or not with 50ng/ml Recombinant Human VEGF 165 (PeproTech, 100-20-2UG) for 24 hours. Total cellular RNA was isolated using the Trizol reagent and extracted through Direct-zol RNA MicroPrep kit (Zymo Research, #R2060).

## Supplementary Material

Supplement 1

Supplement 2

Supplement 3

Supplement 4

Supplement 5

Supplement 6

## Figures and Tables

**Figure 1:**
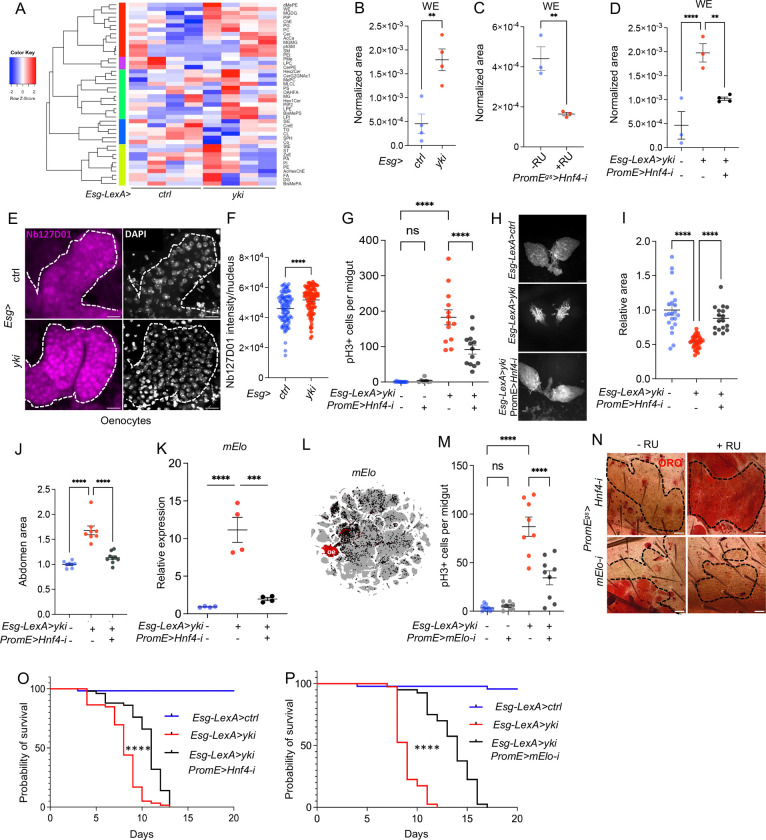
Oenocyte Hnf4 regulates wax ester synthesis and supports Yki-induced tumor growth. (A-B) Heatmap of the normalized total ion intensity of major lipid classes identified in whole bodies from control flies (*Esg-LexA*) or Yki flies (*Esg-LexA/LexAop-yki*^*S3A*^*; PromE-GAL4, Tub-GAL80*^*TS*^/+), with four biological replicates per condition. Scaled colors are presented as the values relative to the average of each row. (B) Untargeted lipidomic profiling using LC-MS/MS of whole-body extracts revealed increased wax ester (WE) levels in Yki flies. (C) WE level in oenocyte-specific knockdown of *Hnf4* using the RU486-inducible *PromE*^*gs*^ driver (+RU) compared to controls (−RU). (D) WE level in control flies, Yki flies, and *Hnf4* knockdown in oenocytes of Yki flies, N = 4 biological replicates. (E-F) Immunostaining of Nb127D01 for nuclear-localized, endogenously tagged Hnf4 (Hnf4-127D01) in oenocytes in controls and in Yki flies. DAPI staining marks nuclei, with dashed lines enclosing oenocytes. N = 8 independent experiments. (G) Quantification of intestinal stem cell (ISC) proliferation, as indicated by phospho-histone H3 (pH3+) cells in control. (H-I) Ovary sizes and quantification. (J) Abdominal bloating. (K) UMAP from whole body (without head) snRNAseq, *mElo* expression is enriched in adult oenocytes. (L) *mElo* expression in oenocytes, normalized to housekeeping gene, in Yki flies and in Yki flies with *Hnf4* knockdown. N = 2 biological replicates from 2 independent experiments. (M) Quantification of pH3+ cell number, N =8 flies. (N) Oil Red O staining in oenocytes of *Hnf4* knockdown or *mElo* knockdown. N = 8 flies. (O-P) Lifespan analysis showing that oenocyte-specific knockdown of *Hnf4* (O) or *mElo* (P) in Yki flies, compared to only Yki or control flies, N = 40. Scale bar shows 20μm. Data are presented as mean ± s.e.m. Statistical significance was determined using Student’s t-test or one-way ANOVA with appropriate post hoc tests, or Log-rank (Mantel-Cox) test. *P < 0.05, **P < 0.01, ****P < 0.0001.

**Figure 1S:**
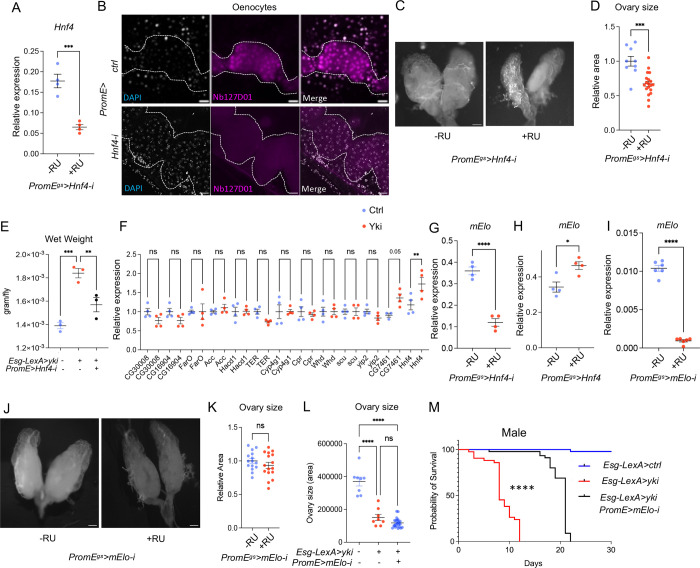
Hnf4 and mElo regulate metabolic and tumor-related phenotypes in Yki flies. (A) Confirmation of *Hnf4* knockdown efficiency via qPCR, N = 2 biological replicates from 2 independent experiments. (B) Immunostaining of Nb127D01 for nuclear-localized, endogenously tagged Hnf4 (Hnf4-127D01) in oenocytes with *Hnf4* knockdown or control flies, with dashed lines enclosing oenocytes. (C-D) Oenocyte-specific *Hnf4* knockdown reduces ovary size compared to control flies, quantification in (D), n = 9 – 15. (E) Wet weight measurement of fly whole body, N = 3 biological replicates. (F) Expression of VLCFA biosynthesis and β-oxidation genes in control and Yki flies. (G-H) *mElo* expression measured by qPCR in oenocyte specific *Hnf4* knockdown or *Hnf4* overexpression, N = 2 biological replicates from 2 independent experiments. (I) Confirmation of *mElo* knockdown efficiency via qPCR. (J-K) Ovary size in oenocyte *mElo* knockdown flies compared to control, quantification in (K). (L) Ovary size measurement in control and Yki flies, N = 8–10 flies. (M) Lifespan analysis in Yki-expressing flies upon oenocyte-specific *mElo* knockdown in male flies. Data represent mean ± s.e.m.; significance assessed using appropriate statistical tests.

**Figure 2:**
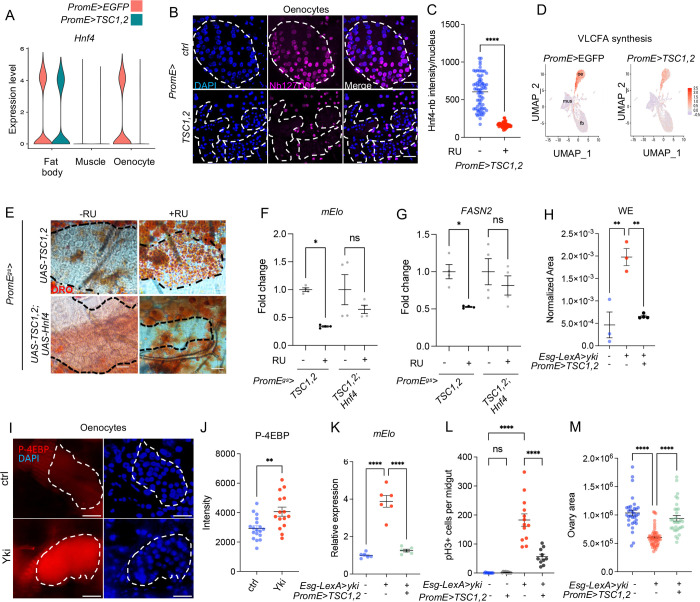
TORC1 regulates Hnf4 activity and lipid metabolism in oenocytes. (A) snRNAseq analysis showing *Hnf4* expression in fat body, muscle and oenocytes upon overexpression of *TSC1,2* in oenocytes. (B-C) Immunostaining of Hnf4-127D01 in oenocytes when *TSC1,2* is overexpressed in oenocytes compared to control. DAPI marks nuclei, with dashed lines enclosing oenocytes, quantification of Hnf4-127D01 signal intensity in the nucleus, N = 8–10 flies. (D) ModuleScore from snRNAseq analysis visualizes VLCFA synthesis gene expression across muscle, fat body and oenocytes, in control and upon oenocyte *TSC1,2* overexpression. (E) ORO staining indicates steatosis in oenocytes induced by *TSC1,2* overexpression, and in *TSC1,2* with *Hnf4* overexpression, N = 8. (F-G) Expression of Hnf4 targets *mElo* and *FASN2* in *TSC1,2* overexpression and in *TSC1,2* with *Hnf4* overexpression. N = 2 biological replicates from 2 independent experiments. (H) Whole body WE level in ISC Yki flies and Yki with oenocyte *TSC1,2* overexpression. (I-J) P-4EBP immunostaining indicates TORC1 activity in oenocytes of Yki flies, quantification of P-4EBP intensity is shown on the right (J), N = 8–10 flies. (K) *mElo* expression in whole body mRNA extracts in Yki flies and Yki flies with *TSC1,2* overexpression in oenocytes, N = 6 biological samples. (L) Quantification of pH3+ cell number, N = 8 – 10 flies. (M) Ovary size measurements are rescued by *TSC1,2* overexpression in Yki flies, N = 15–30 flies.

**Figure 2S:**
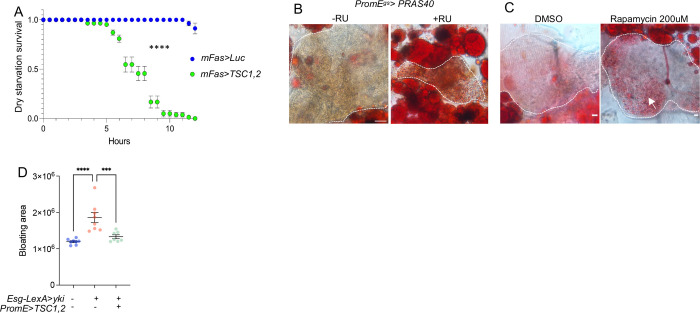
The role of oenocyte TORC1 in regulating lifespan, steatosis and Yki wasting phenotype. (A) Sensitivity to dry starvation in flies overexpressing *TSC1,2* in oenocytes compared to controls. Adult oenocyte specific driver (*mFas-Gal4*) was used [66]. (B-C) ORO staining indicates steatosis in oenocytes with *PRAS40* overexpression, or in rapamycin treatment. (D) Host bloating quantification in fly abdomen in Yki flies and in Yki flies with *TSC1,2* overexpression, compared to control. Dashed lines enclose oenocytes in all immunostaining images. Data represent mean ± s.e.m.; statistical significance determined using Student’s t-test or ANOVA as appropriate.

**Figure 3:**
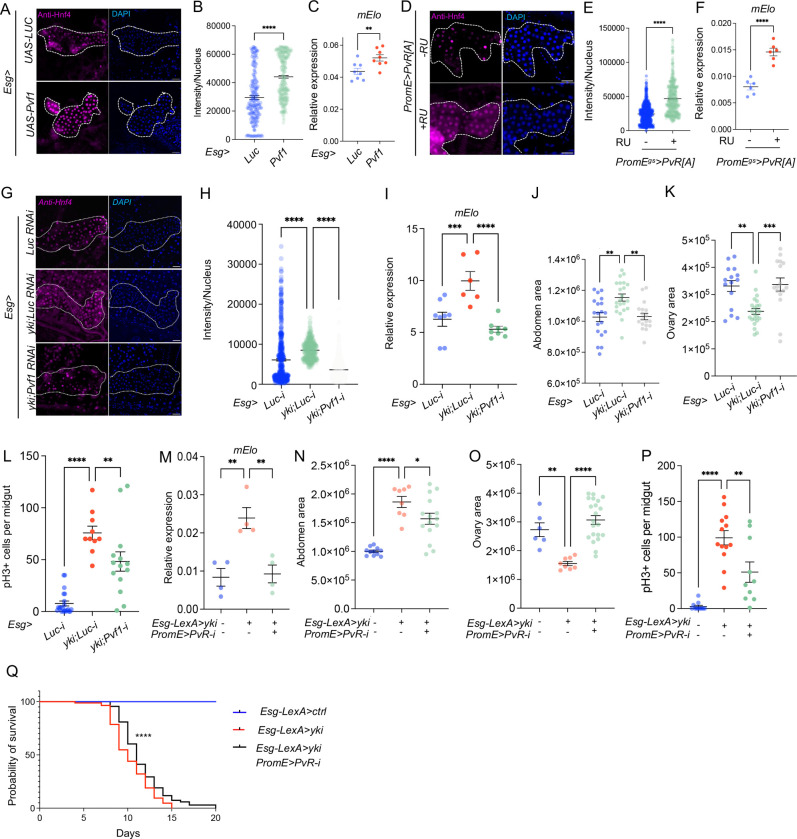
ISCs secreted Pvf1 activates Hnf4 through PvR signaling in oenocytes and affects tumor associated phenotypes. (A-B) Immunostaining of Hnf4-127D01 nuclear localization in oenocytes upon *Pvf1* overexpression in ISCs (*Esg*>*Pvf1*). Dashed lines enclose oenocytes. N = 10 biological replicates. (C) *mElo* expression in oenocytes following ISC-specific *Pvf1* overexpression. N = 4 biological replicates from 2 independent experiments. (D-F) Immunostaining and qPCR analysis reveal Hnf4-127D01 nuclear localization and *mElo* expression in oenocytes overexpressing active *PvR*. (G-H) Immunostaining of Hnf4-127D01 in oenocytes of Yki flies (*Esg*>*yki*^*3SA*^*;Luc-i*) and flies with knockdown of *Pvf1* in Yki flies (*Esg*> *yki*^*3SA*^*;Pvf1-i*). N = 10 biological replicates. (I) *mElo* expression in oenocytes of *Esg*>*yki*^*3SA*^*;Pvf1-i* flies, N = 4 biological replicates from 2 independent experiments. (J-K) Inhibition of *Pvf1* in the gut affects bloating and ovary size in Yki flies, n = 15–20 flies. (L) ISC mitosis (pH3+ cells) in *Esg*>*yki*^*3SA*^*;Pvf1-i* flies, n = 8–10 biological replicates. (M) Yki-induced *mElo* expression. Dual LexA and Gal4 systems to express *yki*^*3SA*^ in ISCs and mediate knockdown of *PvR* in oenocytes (*Esg-LexA*> *yki*^*3SA*^*; PromE*>*PvR-i*). N = 2 biological replicates from 2 independent experiments. (N-P) *PvR* knockdown in oenocytes and its effect on tumor-associated bloating (N), ovary wasting (O), and pH3+ cell number (P), N = 10–20 flies. (Q) Lifespan extension in Yki flies with oenocyte-specific *PvR* knockdown, N = 40 flies. Dashed lines enclose oenocytes in all immunostaining images. Data are presented as mean ± s.e.m.; statistical significance was assessed using Student’s t-test or ANOVA.

**Figure 3S:**
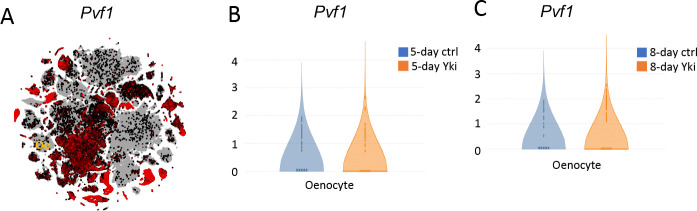
Expression of *Pvf1* in fly whole body and oenocytes in Yki flies. UMAP from whole body (without head) snRNAseq, shows *Pvf1* expression level, oenocyte cluster is marked as “oe”. (B) snRNAseq analysis on oenocyte *Pvf1* expression in control (*Esg*>+) and Yki flies (*Esg*>*yki*^*3SA*^) at 5 days. (C) snRNAseq analysis of oenocyte *Pvf1* expression at 8 days of *yki*^*3SA*^ induction.

**Figure 4:**
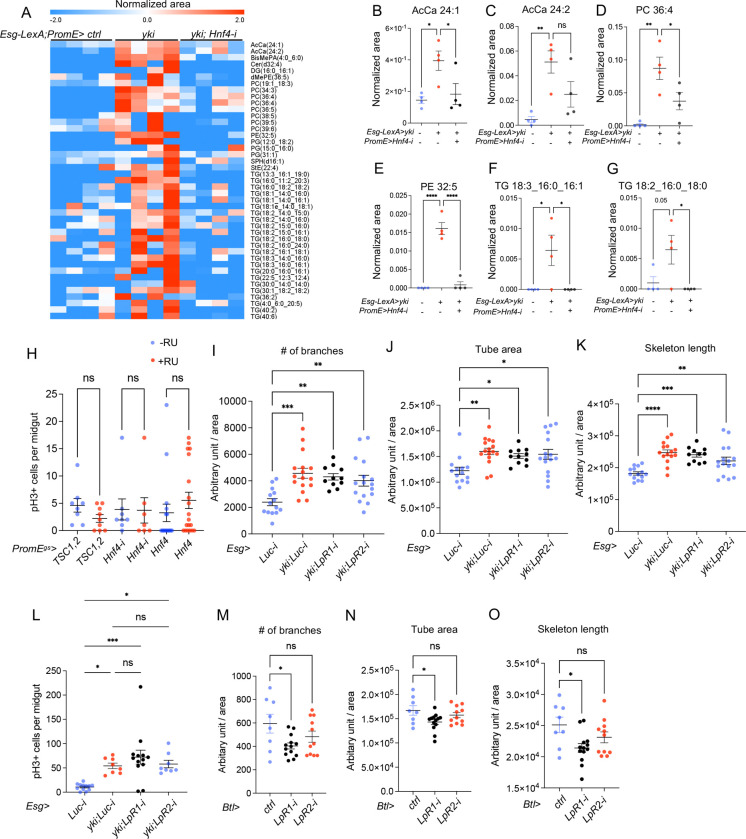
Circulating lipids target trachea but not ISCs to promote tracheal growth. Untargeted lipidomic analysis of fly hemolymph showing lipid species in control, Yki flies, and Yki flies with oenocyte-specific *Hnf4* knockdown (*Esg*>*yki*^*3SA*^*;PromE*>*Hnf4-i*). N = 4 biological replicates. (B-C) Levels of very-long-acylcarnitines (AcCa 24:1 and AcCa 24:2) in Yki flies with or without *Hnf4* knockdown in oenocytes. (D-E) Levels of circulating phospholipids, including PC 36:4 PE 32:5, across experimental conditions. (F-G) Levels of circulating TG 18:3_16:0_16:1 and TG 18:2_16:0_18:0 in the fly hemolymph. (H) Counts of pH3+ cells in the midgut of flies with oenocyte-specific *TSC1*,2 overexpression or *Hnf4* knockdown or overexpression. N = 8–20 flies. (I-K) Number of tracheal branches, tube area, and skeleton length in Yki flies with ISC-specific knockdown of *LpR1* and *LpR2*. (L) pH3+ cell counts in ISCs of Yki flies with ISC-specific knockdown of *LpR1* and *LpR2*. (M-O) Effects of trachea-specific knockdown of *LpR1* and *LpR2* using the *Btl-Gal4* driver on trachea parameters, including number of branches, tube area, and skeleton length, compared to control flies (*Btl*>*ctrl*). N = 8–12 flies. Dashed lines enclose oenocytes in all relevant images. Data are presented as mean ± s.e.m.; statistical analysis was performed using Student’s t-test or ANOVA as appropriate.

**Figure 4S:**
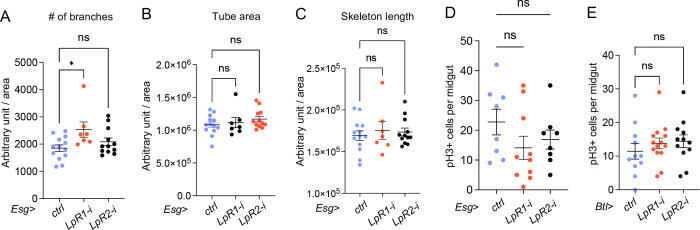
Effects of ISC-specific *LpR1* and *LpR2* knockdown on tracheal morphology and ISC proliferation. (A-C) Trachea parameters, including number of branches, tube area, and skeleton length, in flies with ISC-specific knockdown of *LpR1* and *LpR2* using *Esg* driver, compared to control (*Esg*>*ctrl*). N = 7–11 flies. (D) Counts of pH3+ cells in the midgut of flies with ISC-specific knockdown of *LpR1* and *LpR2*, compared to controls. (E) Counts of pH3+ cells in the midgut of flies with trachea-specific knockdown of *LpR1* and *LpR2*, compared to controls. N=8–10 flies. Data are presented as mean ± s.e.m.; statistical analysis was performed using Student’s t-test or ANOVA as appropriate.

**Figure 5:**
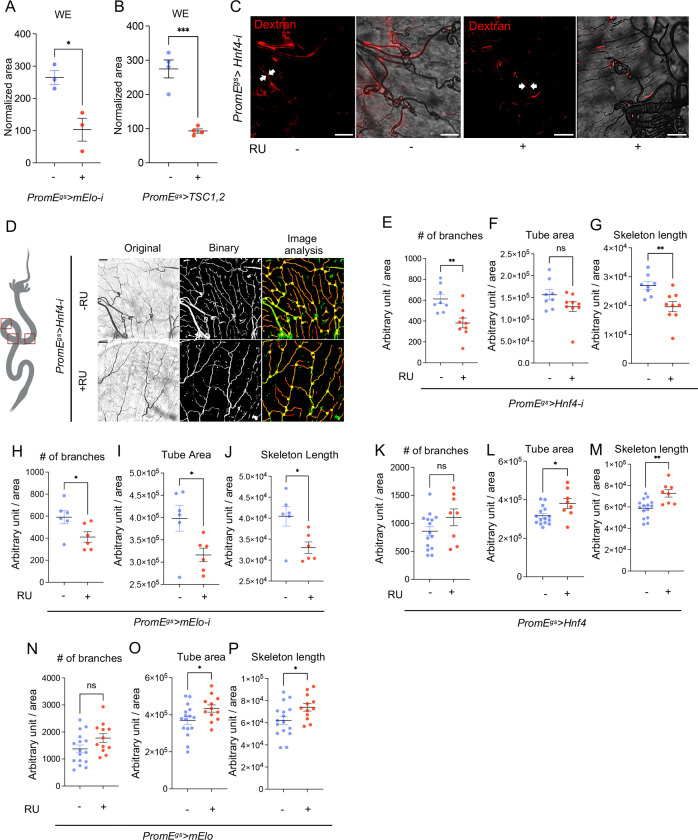
Hnf4-mElo axis regulates tracheogenesis in the midgut. (A-B) Whole-body wax ester (WE) levels in flies with oenocyte-specific *Hnf4* knockdown (*PromE*^*gs*^>*Hnf4-i*), *mElo* knockdown (*PromE*^*gs*^>*mElo-i*), or *TSC1,2* overexpression (*PromE*^*gs*^>*TSC1,2*). N = 3–4 biological replicates (C) Transepithelial barrier function of the trachea on the midgut, assessed using red dextran injection, in flies with oenocyte-specific *Hnf4* knockdown. N = 8 flies. (D-G) Trachea morphology on the midgut (R2-R4), including branch number and skeleton length normalized to gut surface area, in flies with *Hnf4* knockdown in oenocytes. N = 8 flies. (H-J) Branch number, tube area, and skeleton length of the midgut trachea in flies with oenocyte-specific *mElo* knockdown. N = 6–8 flies (K-P) Number of branches, tracheal tube area and skeleton length of the midgut in flies overexpressing *Hnf4* or *mElo* in oenocytes (*PromE*^*gs*^>*Hnf4* or *PromE*^*gs*^>*mElo*). N = 8–15 flies.

**Figure 5S:**
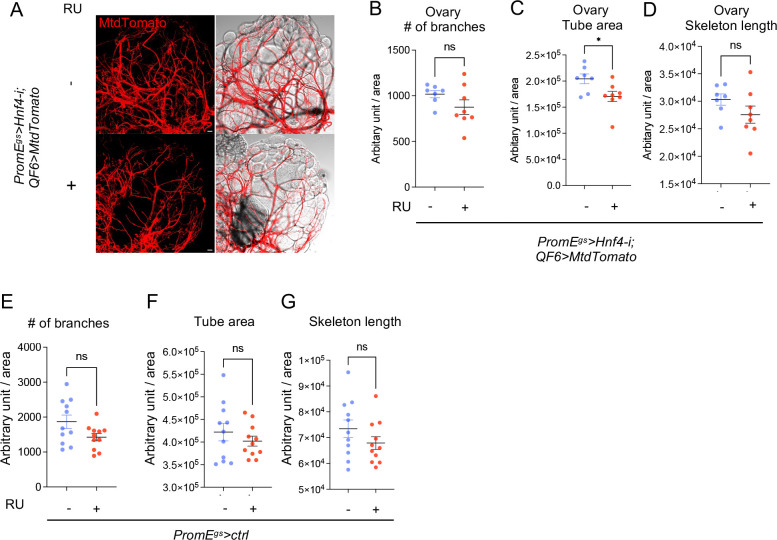
Effect of oenocyte Hnf4 on ovary trachea. (A-D) Tracheal branch number, tube area and total skeleton length of the ovary in flies with *Hnf4* knockdown in oenocytes. N = 8 flies. (E-G) Comparison of gut trachea morphology in wild type flies (*PromE*^*gs*^>+) with and without RU feeding. N = 12 flies. Dashed lines enclose oenocytes in all relevant images. Data are presented as mean ± s.e.m.; statistical analysis was performed using Student’s t-test or ANOVA as appropriate.

**Figure 6:**
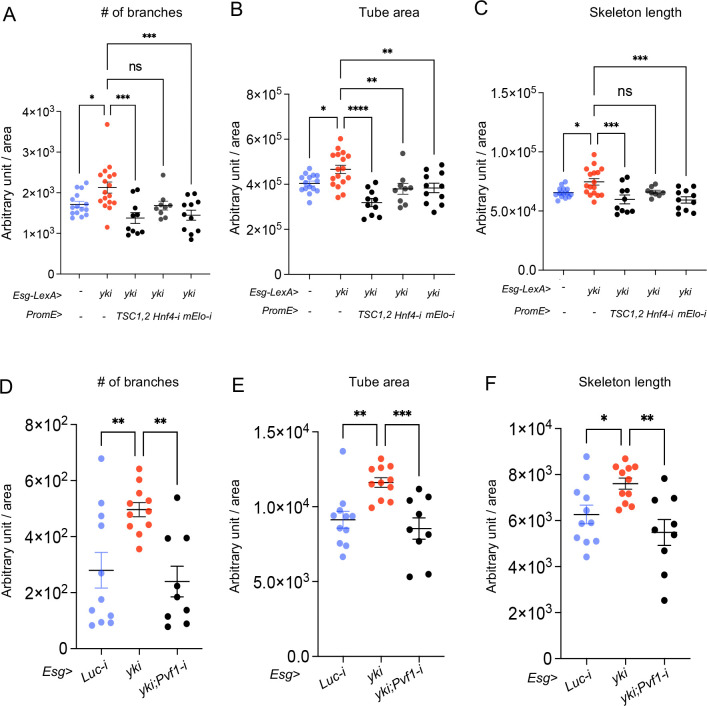
Hnf4-mElo axis in oenocytes mediates lipid metabolism and tracheogenesis induced by Yki tumors. (A-C) Number of tracheal branches, total tube area, and total tube length in the midgut of Yki flies with oenocyte-specific *TSC1,2* overexpression, *Hnf4* knockdown, or *mElo* knockdown. N = 9–17 flies. (D-F) Tracheal morphology measurements, including number of branches and skeleton length, in flies with ISC-specific knockdown of *Pvf1* combined with *yki* overexpression (*Esg*>*yki*^*3SA*^*;Pvf1-i*). N = 9–11 flies. Dashed lines enclose oenocytes in all relevant images. Data are presented as mean ± s.e.m.; statistical analysis was performed using Student’s t-test or ANOVA as appropriate.

**Figure 6S:**
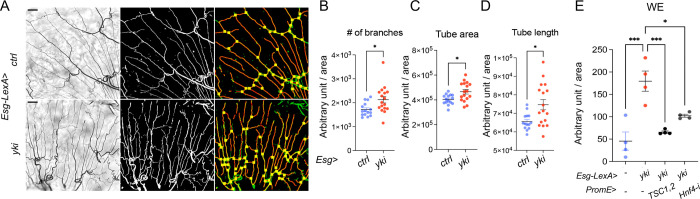
Hnf4-mElo axis in oenocytes mediates lipid metabolism and tracheogenesis induced by Yki tumors. (A-D) Tracheal morphology parameters, including number of branches, total tube area, total tube length, and skeleton length, in Yki midgut (*Esg*>*yki*), compared to wild-type controls (*Esg*>*ctrl*). N = 16–17. (E) Whole-body wax ester (WE) levels in Yki flies with oenocyte-specific *Hnf4* knockdown (Esg-LexA>LexAop-yki^3SA^; *PromE*>*Hnf4-i*) or *TSC1,2* overexpression (Esg-LexA>LexAop-yki^3SA^; *PromE*>*TSC1,2*), measured to investigate the role of lipid metabolism in trachea growth. N = 4 biological replicates.
